# Natural bio-based monomers for biomedical applications: a review

**DOI:** 10.1186/s40824-021-00208-8

**Published:** 2021-04-01

**Authors:** Mallinath S. Birajdar, Haejin Joo, Won-Gun Koh, Hansoo Park

**Affiliations:** 1 Department of Integrative Engineering, Chung-Ang University, Seoul, Republic of Korea; 2 Department of Chemical and Biomolecular Engineering, Yonsei University, Seoul, Republic of Korea

**Keywords:** Natural bio-based monomers, Itaconic acid, Succinic acid, Citric acid, Hyaluronic acid, Glutamic acid

## Abstract

**Abstract:**

In recent years, synthetic and semi-synthetic polymer materials have been widely used in various applications. Especially concerning biomedical applications, their biocompatibility, biodegradability, and non-toxicity have increased the interest of researchers to discover and develop new products for the well-being of humanity. Among the synthetic and semi-synthetic materials, the use of natural bio-based monomeric materials presents a possible novel avenue for the development of new biocompatible, biodegradable, and non-toxic products. The purpose of this article is to review the information on the role of natural bio-based monomers in biomedical applications. Increased eco-friendliness, biocompatibility, biodegradability, non-toxicity, and intrinsic biological activity are some of the attributes which make itaconic, succinic, citric, hyaluronic, and glutamic acids suitable potential materials for biomedical applications. Herein, we summarize the most recent advances in the field over the past ten years and specifically highlight new and interesting discoveries in biomedical applications.

**Graphical abstract:**

Natural origin acid-based bio-monomers for biomedical applications 
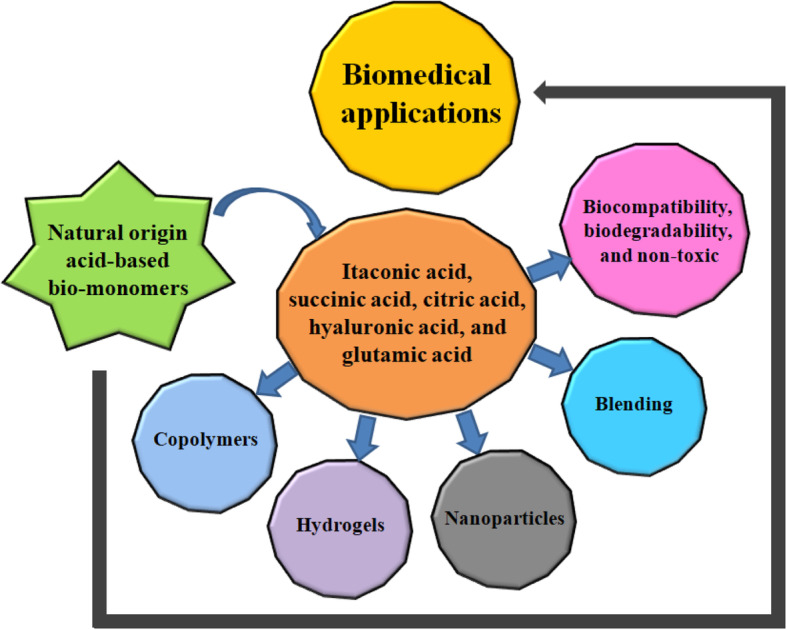

## Introduction

Natural bio-based monomers are sourced from nature (plants or animals) and are widely used for biomaterials production due to their biodegradability, biocompatibility, microstructure, morphology, modifiable mechanical properties, and versatility. A biomaterial is defined as any natural or synthetic material engineered to interact with biological systems to direct medical treatment [1]. Biomaterials must be biocompatible, biodegradable, and non-toxic, meaning that they must be able to perform their function with an appropriate host response [2]. To meet the needs of the biomedical community, materials composed of almost anything (from metals and ceramics to glass and polymers) have been discovered. Natural bio-based monomers are particularly attractive for biomedical applications as they possess preferred criteria, particularly biocompatibility, biodegradability, and high porosity; they can undergo a diverse range of chemical and physical modifications specific to tissue regeneration; and they possess biological properties that are desirable for biomedical applications [3, 4]. Natural bio-based monomers and biopolymers commonly used in biological scaffold production include collagen, fibrin, fibrinogen, platelet-rich plasma, alginate, gelatin, albumin, and hyaluronic acid. A major advantage of bioscaffolds from natural bio-monomers is that they are more likely to encourage cell growth [5].

While natural polymers like chitosan, alginate, hyaluronic acid, and collagen have been used biomedically for thousands of years, research into biomedical applications of synthetic degradable polymers is relatively new, having started in the 1960s [6–9]. In the last five decades, although successes have been many, massive challenges still exist in both the basic and translational elements of natural polymer material design. From a basic science perspective, the efficiency to modulate biomaterial chemistry for conveying unique material properties is endless yet requires significant time and resources to complete the research. As natural bio-based monomers and biopolymer materials are applied in the clinical setting, many issues arise that have not been adequately identified and addressed in previous in vitro and in vivo experiments. The host response to both tissue engineering and drug-delivery devices depends on the chemical, physical, and biological properties of the polymer materials. To better address the many issues in biomaterials design and to hasten progress, biomaterials scientists have fundamentally changed their outlook on research. Especially in the last decade, there has been a shift in the paradigm from investigators working independently on narrow research goals to collaborative teams that facilitate solving greater objectives. By combining researchers with expertise in biology, chemistry, materials, engineering, and clinical practice, biomaterials research has been able to advance more rapidly in the past few years [10–18].

In the design of suitable biocompatible, biodegradable, and non-toxic biomaterials, many important properties must be considered. These materials should not produce a continuous inflammatory reaction, possess a degradation time coinciding with their function, have suitable mechanical properties for their intended use, produce non-toxic biodegradation products that can be readily resorbed or excreted, and include appropriate permeability and processability for designed application [19]. These properties are greatly affected by some of the features of biocompatible and degradable polymeric biomaterials, including (but not limited to) their chemistry, molecular weight, hydrophobicity, surface charge, water adsorption, degradation, and erosion mechanism. Because of the wide-ranging use of polymeric biomaterials, a single ideal polymer or polymeric family does not exist. Instead, some natural bio-based monomeric materials are available to researchers that can be engineered and chemically and/or physically modified to best match the specifications of the material’s desired biomedical function. Among them, graft copolymerization is an easy chemical method to modify the structure of natural monomers/polymers as it makes them attractive biomaterials in biomedical applications. Since, polymers and their modified forms have been used in diverse fields.

Herein, we focus on the numerous advancements made in the development of biocompatible, biodegradable, and non-toxic biomaterials by using natural bio-based monomers for biomedical applications over the past decade.

## Natural bio-based monomers–An emerging alternative solution

Over the past few years, there has been much attention on the use of natural bio-based monomers in different application fields. Natural bio-based monomers derived from plants, microorganisms, and animals’ resources have several advantages such as abundant availability, biocompatibility, biodegradability, and non-toxicity [8, 20, 27, 32, 42]. In biomedical applications, the use of engineered or chemically and/or physically modified natural bio-based monomers is a new concept that has been developed in recent years. This was brought about by the recognition that their unique properties can be applied to different application areas such as antimicrobial coatings [20], surfactant and detergent manufacturing [31], chelating agents [36], antioxidant manufacturing [38], and adsorbents [46], which have recently become popular. The natural bio-based acidic monomers are of great interest due to which derived from plants, microorganisms, and animals’ resources have several advantages such as abundant availability, biocompatibility, biodegradability, non-toxicity, and consisting of naturally derived building blocks that are chemically reactive for the modifications of polymers when they are specifically applied for biomedical, pharmaceutical, or other bioengineering applications.

## Itaconic acid

Itaconic acid is a promising bio-based monomer material and can be obtained on a large scale using fermentation processes. It was discovered by Baup in 1836 from the pyrolysis of citric acid [53] but was only reported as a biological product synthesized by *Aspergillus itaconicus* in 1932 [54]. Its non-toxicity, biocompatibility, biodegradability, chemical reactivity, and microbe resistance have given it huge potential in a broad range of scientific uses in the biomedical, food, agricultural, pharmaceutical, and other industries [55].

### Biomedical applications of Itaconic acid

The applications of bio-based itaconic acid monomer are well documented, but its biomedical applications sin engineered or chemically and/or physically modified polymers have not yet been completely reviewed. Indeed, the use of their modified forms has spread to the dental, ophthalmic, and drug-delivery areas [55]. From the available literature, it can be deduced that the structure, type of cross-linker, functionalizing mechanism, concentration, and selection and location of the functionalized groups are to a large extent influenced by natural bio-based monomers for engineering, chemical, and physical methods. The antimicrobial activity, drug carrier ability, wound-healing ability, biocompatibility, biodegradability, and chemical reactivity of itaconic acid is well documented (Table 1), although its modified forms and their uses have yet not been fully elucidated.

**Table 1:** Characteristics of some natural bio-based monomers used in biomedical applications

Bio-based monomers	Source	Characteristics	References
Itaconic acid	*Aspergillus itaconicus*	Antimicrobial activity, non-toxic, biocompatible, biodegradable, chemical reactivity, surfactant forming ability, hydrophilic activity, wound-healing activity, coating forming ability, water uptake ability, drug carrier ability, and hydrogel-forming ability	[20–26]
Succinic acid	*Actinobacillus succinogenes*, *Anaerobiospirillum*, and *Mannheimia succiniciproducens*	Biocompatible, biodegradable, non-toxic, chemical reactivity, food additives ability, food flavoring ability, surfactant/detergent extender/foaming ability, drug carrier ability, pH control ability, antimicrobial activity, and corrosion prevention ability	[27–31]
Citric acid	Citrus fruits and *Aspergillus niger*	Biocompatible, biodegradable, non-toxic, excellent chelating property, anti-odor property, chemical reactivity, pH control ability, food additives ability, food flavoring/preservative ability, and drug carrier ability	[32–37]
Hyaluronic acid	Rooster comb, human (umbilical cord, joint (synovial) fluid, vitreous body, dermis, epidermis), bovine nasal cartilage, and rabbit brain and heart.	Biocompatible, biodegradable, non-toxic, viscoelastic property, excellent gelling property, anti-inflammatory property, wound-healing activity, excellent cosmetic property, and drug carrier ability	[8, 38–41]
Glutamic acid	*Bacillus subtilis* and *Bacillus licheniformis*	Biodegradable, biocompatible, non-toxic, excellent chelating property, heavy metal removal ability, cosmetic property, drug carrier ability, hydrophilic activity, anionic property, thickener property, aging inhibitor ability, and use as an additive	[42–52]

Sood and co-workers [56] reported the preparation of an itaconic acid-grafted carboxymethyl cellulose-cl-poly (lactic acid-co-itaconic acid) hydrogel via facile graft copolymerization under microwave irradiation. The prepared hydrogel showed controlled drug release, and antimicrobial studies showed it to be more effective against gram-positive *Staphylococcus aureus* than gram-negative *Escherichia coli*. The antibacterial activity of the hydrogel was due to monomers grafted on the surface of polymer causing the degradation of the membrane structure of the bacterial cells, thereby damaging the cell wall and allowing the leakage of proteins and other intracellular constituents that ultimately causes cell death [57, 58]. The most accepted view is that antimicrobial activity increases with an increase in itaconic acid monomer concentration.

Gupta and co-workers [59] reported the development of itaconic acid-grafted poly (acrylamide-co-itaconic acid) cotton fabric through graft copolymerization in an aqueous medium. The developed fabrics showed biocidal action against *E. coli*, thus showing great potential for use as antiseptic dressings and bandages, which are in high demand for biomedical applications. Previously, it has been reported that the presence of itaconic acid monomer enhances the microbe resistance of hydrogels [60], and it has also been reported that the presence of itaconic acid enhances the wrinkle resistance of silver nanoparticles-loaded poly (acrylamide-co-itaconic acid)-grafted cotton fabric [61]. For the first time, Yin and co-workers [62] reported the synthesis of itaconic acid-grafted carboxymethyl chitosan (PICMCS) and its nanoparticles via free-radical polymerization. PICMCS and PICMCS nanoparticles showed good stability and better cytocompatibility toward L929 cells than carboxymethyl chitosan due to the following effective advantages: (1) decreasing the cationic damage to the cell membrane due to consumption of the free amino groups on the CMCS molecules by the grafting reaction with itaconic acid; (2) increasing the negative charge contributed by carboxyl groups on itaconic acid, thereby leading to less interaction with the negatively charged cell membrane and a great improvement in the stability of PICMCS nanoparticles due to the negatively charged surface; and (3) extending the longevity of nanoparticles in the blood circulatory system because of improved hydrophilicity after grafting itaconic acid that can protect the nanoparticles from being captured by the reticuloendothelial system [62, 63]. Overall, the grafting of itaconic acid monomer largely improves the stability and cytocompatibility of the resulting nanoparticles.

Işıklan and co-workers [64] reported the synthesis of itaconic acid-grafted alginate-g-poly (itaconic acid) (NaAlg-g-PIA) copolymer and microspheres via graft polymerization. The developed graft copolymer and microspheres showed a pH-responsive nature and sustained drug release via stepwise-release behavior due to the double ionization of itaconic acid monomer at different pH values. The stepwise-release behavior of drugs depends not only on the nature of the medium but also on the nature of the material. It has been previously reported that double ionization of itaconic acid at different pH values provides the stepwise-release behavior of specially adsorbed drugs by controlling the pH of the medium [65]. Moreover, it has also been reported that the presence of itaconic acid monomer bestows chelate formation in certain cases due to the double ionizing ability of the itaconic acid carboxylic groups [66].

Pourjamal et al. [67] reported the synthesis of itaconic acid-grafted superabsorbent nanohydrogels of poly (N-isopropyl acrylamide-co-itaconic acid) via free-radical graft copolymerization. The synthesized nanohydrogels showed high swelling capacity, pH sensitivity, and sustained drug release, and thus are suitable for biomedical applications. Itaconic acid is one of the best bio-based monomers because it has two carboxylic groups that help impart better solubility and water absorption to the nanohydrogels. It has been previously reported that the swelling capacity of nanohydrogels is increased by increasing the amount of itaconic acid monomer, and the higher hydrophilicity of itaconic acid could be responsible for increasing the swelling capacity of hydrogels [67]. It has also been reported that drug release can be made faster by increasing the amount of itaconic acid monomer present in the hydrogels [68]. Overall, the grafting of bio-based itaconic acid monomer largely improves the non-toxicity, biocompatibility, swelling capacity, pH sensitivity, hydrophilicity, and drug-carrying capacity of hydrogels and graft polymers.

## Succinic acid

Succinic acid monomer was discovered by Georgius Agricola in 1546 [31]. It is a C-4 dicarboxylic acid that is nowadays considered to be one of the most promising bio-based monomer produced by microbial fermentation [69]. However, the industrial potential for succinic acid fermentations was recognized as early as 1980 [70]. The bio-based succinic acid monomer has been used in the food industry since Robert Koch proved that it has a positive influence on human metabolism without the risk of accumulation in the human body [71]. Derived from various microorganisms and agricultural carbohydrates, it is non-toxic, biocompatible, and biodegradable, and so is widely used in the development of biomedical products, food additives, pharmaceutical products, surfactants, detergents, microbe-resistant products, green solvents, and biodegradable plastics [31].

### Biomedical applications of succinic acid

The industrial applications of bio-based succinic acid monomer have been well described, although based on a literature survey, the biomedical applications of chemically and/or physically modified succinic acid monomer in polymer biomaterials have not yet been fully reviewed. Based on previous studies, it can be deduced that the selection of functional groups; their structure, concentration, and location; and the type of cross-linker have a large influence on the engineering, chemical, and/or physical methods applied to natural biopolymers.

Previously, it has been reported that bio-based succinic acid monomer can be used to synthesize various chemicals and products [27]. Most recently, Yang and co-workers [72] prepared three novel polymeric surfactants with succinic acid monomer by the reaction of maleic anhydride monomer and polyethylene glycol (PEG), which are bio-based materials, environmentally friendly, and biocompatible surfactants. The synthesized succinic acid-based polymeric surfactants exhibited excellent surface activities with great efficiency in decreasing the surface tension of water. Polymeric surfactants have a characteristic molecular structure consisting of a hydrophobic chain together with a hydrophilic portion. In comparison to traditional surfactants, they exhibit excellent properties such as dispersion, cohesion, thickening, and emulsification. Due to their unusual properties, they are widely used as dispersers, flocculants, and rheology modifiers [73–77]. With these characteristics in mind, Yang and co-workers [72] envisioned a new surfactant, containing two sulfate groups and long chains of (−CH_2_CH_2_O–) that can not only further enhance solubility but also provide a steric effect and an electrostatic repulsive force for dispersion stabilization.

Bondar et al. [78] developed succinic acid-conjugated Pluronic copolymers via the reaction of bio-based succinic acid monomer and Pluronic micelles L61 and L121, both of which are biologically non-toxic and biocompatible. The succinic acid-conjugated Pluronic copolymers exhibited excellent interaction with cell plasma membranes and low cytotoxicity. They evaluated the mechanism of interaction of amphiphilic polymers with living cells and showed that succinic acid-Pluronic conjugates can be used as safe and efficient modulators of intracellular drug delivery. Hence, they hypothesized that Pluronic L61 and L121 conjugated with succinic acid modulates their interaction with the cellular membrane.

In a previous study, Carnahan and co-workers [79] prepared a variety of dendrimer adhesives consisting of generations 1, 2, and 3 (G1, G2, and G3) associated with succinic acid monomer, PEG, and glycerol to repair corneal wounds. These dendrimer adhesives were developed via photopolymerization in situ. The specific polymer used was a dendritic linear copolymer composed of succinic acid monomer, PEG, and glycerol. Once prepared, these dendritic macromolecules were further functionalized with photo-cross-linkable groups. The gels possessed sufficient tissue adhesive properties to seal corneal lacerations. These polyester ether ABA-triblock copolymers have expanded the polymers available for study. Tailoring the linear and dendritic blocks can afford macromolecules with unique and interesting chemical, physical, and mechanical properties. Moreover, their structures are intricately designed in such a way that the C_2_ position of glycerol serves as a branching point, while succinic acid serves both as the core and the linker between the branching points. The authors suggest that these characteristics are likely to facilitate the design, development, and use of new biomaterials for specific biomedical applications.

Mitra et al. [80] prepared a novel 3D scaffold of chitosan and collagen separately using succinic acid monomer as a cross-linker and discussed the chemistry behind the cross-linking and the improvement in mechanical and thermal properties of the cross-linked material in detail. They explicitly demonstrated that succinic acid acts as a suitable cross-linker for the preparation of a biocompatible and non-toxic scaffold comprising chitosan and collagen with appreciable mechanical properties. Moreover, the interaction of succinic acid monomer with chitosan and collagen was identified as non-covalent. In general, the mechanical properties of any scaffold material depend on the interaction between the cross-linker/stabilizer and parent molecules (here it is chitosan and type-I collagen).

It has been well documented previously that covalent interactions predominate in the bonding of cross-linkers, which ultimately prevents the biomaterial from attaining the desired mechanical strength [81–86]. Thus, to avoid problems associated with obtaining suitable mechanical properties, biocompatibility, and non-toxicity of biomaterials, the parent molecules should be cross-linked with a suitable cross-linker through non-covalent interactions. For example, succinic acid is a natural bio-based monomeric cross-linker and also an intermediate in the Krebs cycle. Due to this extraordinary cross-linking ability, it has been suggested that succinic acid can be used as a natural bio-based monomeric cross-linker for the development of biocompatible, non-toxic, and strong biomechanical scaffolds.

For the first time, Bahama and co-workers [87] synthesized unique elastic polyesters via the simple free catalytic polyesterification of multifunctional monomers derived from succinic acid, glycerol, and azelaic acid. The resulting polyesters exhibited a unique characteristic in that they become soft and pliable after removal from the oven and then harden at room temperature. The authors describe the preparation of easily processable thermoplastic materials that have a low environmental impact, i.e., polymers derived from renewable chemicals. Succinic and azelaic acids monomers are a good choice as they are made from renewable resources and are suitable for biomedical applications because of their antibacterial properties [88, 89]. According to the US Food and Drug Administration (FDA), succinic acid monomer is safe and is used as a flavor enhancer, a pH control agent in food products, and as an ingredient in toothpaste [88]. Glycerol is a trifunctional compound that can be combined with dicarboxylic acids to produce polyesters, and the US FDA has approved glycerol for medical applications, polymers of glycerol and diacids have garnered considerable interest for the development of bioresorbable materials [90]. By combining all of these properties, Bahama et al. [87] successfully prepared the novel and unique elastic polyesters for the first time. Due to these unique characteristics, the authors suggested that not only can succinic acid be used as a simple monomer for the synthesis of elastic polyesters but it can also improve the chemical and physical properties of polyesters by its addition as a bio-based monomer. Hence, succinic acid-modified biocompatible elastomers can be used for future biomedical applications.

## Citric acid

The industrial-scale production of citric acid was first started in 1890 by the Italian citrus fruit industry [36]. However, the microbial production of citric acid did not become important until World War I disrupted Italian citrus exports [91]. In 1917, American food chemist James Currie discovered citric acid production by *Aspergillus niger*, and after two years the pharmaceutical company Pfizer started using this technique for its industrial production [92]. Citric acid is a multifunctional, non-toxic, biocompatible, and biodegradable natural organic compound that is involved in the Krebs cycle. It is widely used in the chemical industry, food industry, cleaning products, and biomaterials production industry.

Industrial and other applications of citric acid have been well presented. However, as per our literature survey, the biomedical applications of citric acid modified chemically and/or physically or citric acid cross-linked polymer biomaterials have not yet been fully reviewed in one review article. Therefore, it is necessary to review the modified forms or citric acid cross-linked polymers. From the findings in some previous reports, it can be concluded that the structures, selection of monomers, concentration, and the type of cross-linker and functional groups have a marked effect on natural bio-based materials via chemical and/or physical modification.

### Biomedical applications of citric acid

Liu and co-workers [93] prepared polyurethane prepolymer by grafting citric acid and chitosan onto polyurethane through covalent immobilization. The prepared materials had improved biocompatibility and antibacterial properties. Citric acid is a natural bio-based monomer/smaller organic compound that is a weak acid, as well as being non-toxic, biocompatible, and biodegradable. Hence, it is widely used as an anticoagulant, anti-odor agent, and food additive/flavoring/preservative agent, as mentioned in Table 1. It has been previously reported that the chemical modification, grafting, physical blending, and cross-linking of some natural and synthetic polymers with citric acid monomer can improve the biocompatibility, biodegradability, and non-toxicity of the modified forms, which is useful for biomedical applications [94–97]. Therefore, it has been suggested that citric acid can be used as an excellent bio-based monomeric material/compound to modify or cross-link with natural, synthetic, and semi synthetic polymers.

Recently, Liu et al. [98] developed a novel chitosan-citric acid film via graft copolymerization. The citric acid-modified film showed excellent hydrophilicity, biocompatibility, biomineralization promotion, and moisture-retaining capacity. Compared with other substances, the molecular composition and spacer arm of the citric acid monomer bestows various functions such as balancing of the hydrophilicity of biomaterials; participating in hydrogen bonding interactions; improving biodegradability, biocompatibility, and non-toxicity; suppressing the bitterness of drugs; and improving the ability of deposition of inorganic ions due to an increase in the number of carboxyl groups [98–100]. It has also been suggested that introducing the carboxyl groups from the citric acid monomer to natural or synthetic polymers can improve the hydrophilicity, biocompatibility, and mineralization ability of the biomaterials.

Jiang et al. [101] developed a new non-toxic cross-linking method for electrospun zein fibers using citric acid as a non-toxic cross-linker that can provide electrospun protein fibers with the water stability required for biomedical applications. The citric acid cross-linked electrospun zein fibers showed improved morphological stability of the fibers, thereby provided better attachment, spreading, and proliferation of NIH 3 T3 fibroblast cells. Although more successful cases could be listed, blending still has several limitations. Under some circumstances, dissolving certain proteins and polymers in one non-toxic solvent that is suitable for electrospinning is challenging. Besides, some properties of blended polymers, such as high hydrophobicity or poor degradability, may cause undesirable changes in the surface properties and biodegradability of the final electrospun protein nanofibers. Hence, chemical modification such as cross-linking has been used as an alternative to polymer blending. However, most cross-linking agents (e.g. glutaraldehyde, formaldehyde, carbodiimide, epichlorohydrin, and sodium metaphosphate) that have been used to cross-link electrospun protein nanofibers increase the toxicity of the biomaterials, are expensive and inefficient in providing the desired improvement in water stability of fibers and bestow low cross-linking ability [102–113]. Recently, attempts have been made to use carboxylic acids such as citric acid to cross-link and improve the mechanical properties and stability of biomaterials without compromising the cytocompatibility [102, 114]. Cross-linking biomaterials with citric acid provides pendant functionality and allows the formation of ester bonds, thereby leading to better hemocompatibility and increased availability of binding sites for bioconjugation [115]. Citric acid is cytocompatible with biosystems because of the intermediate products in the metabolic path, and so is appropriate for modifying the biomaterials.

De’Nobili and co-workers [116] developed citric acid-based alginate edible films using glycerol (plasticizer) for antioxidant food preservation. The developed citric acid-based films have shown excellent antioxidant properties. Citric acid monomer is a weak organic acid naturally found in many fruits, vegetables, and microorganisms. It acts as a synergist by promoting the activity of proper antioxidants due to its metal chelator activity, binding heavy metals (Table 1). Hence, the presence of citric acid together with L-(+)-ascorbic acid in edible films can be useful for antioxidant effect at interfaces. However, citric acid and ascorbic acid can affect each other when both are compartmentalized in the edible film network, and thus the half-life of the active antioxidant interface can be shortened. Hence citric acid, which improves the activity of proper antioxidants like ascorbic acid through its metal chelator ability, can be used together with ascorbic acid for the development of antioxidant alginate-based edible films. Therefore, it has been suggested that citric acid is a suitable bio-based monomer for modifying biomaterials for biomedical applications.

Previously, Mehdizadeh et al. [117] synthesized a new family of injectable citrate-based mussel-inspired bioadhesives via a one-step polycondensation reaction without using toxic reagents for applications in hemostasis and sutureless wound closure. The developed bioadhesive materials showed 2.5 to 8.0 folds stronger wet tissue adhesion strength over clinically used fibrin glue, demonstrated controlled degradability and tissue-like elastomeric mechanical properties, and exhibited excellent cytocompatibility both in vitro and in vivo. Developing surgical adhesive biomaterials with strong wet tissue adhesion, controlled degradability, and mechanical properties, and excellent biocompatibility is a significant challenge, and citric acid could be used to improve these properties.

Citric acid, a non-toxic metabolic product in the Krebs cycle, is key in establishing the methodology for developing citrate-based biodegradable polymers, including poly (diol citrates), cross-linked urethane-doped polyesters, and poly (alkylenes maleate citrates) for biomedical applications [35, 90, 118–124]. It is mainly used to facilitate biodegradable ester-bond formation in biomaterials while enhancing hemocompatibility, biocompatibility, degradability, non-toxicity, and hydrophilicity of the polymers and providing pendant binding sites for bioconjugation to confer additional functionality such as optical properties. As well as the development of injectable citrate-based mussel-inspired bioadhesives [117], citric acid has been used to make not only degradable polyesters with PEG but also provides valuable pendant reactive carboxyl group to conjugate dopamine or L-DOPA. Thus, using highly reactive multifunctional non-toxic citric acid enables a one-step synthesis to prepare biodegradable polyesters with pendant catechol functionalities through a one-step polycondensation reaction. Overall, the grafting, conjugation, chemical cross-linking, and physical blending of bio-based citric acid monomer can largely improve the non-toxicity, biocompatibility, biodegradability, hydrophilicity, and wound-healing capacity for the modified polymers, which could be useful for biomedical applications.

## Hyaluronic acid

Hyaluronic acid was originally isolated by Meyer and Palmer in 1934 and has shown significant promise as a biomaterial. In the same year, Meyer and Palmer wrote in the Journal of Biological Chemistry about an unusual polysaccharide with an extremely high molecular weight isolated from the vitreous of bovine eyes [125]. Hyaluronic acid is a linear polyanionic bio-polysaccharide consisting of alternating units of N-acetyl-D-glucosamine and glucuronic acid making it a member of the glycosaminoglycan family, distributed widely throughout connective, epithelial, and neural tissues [126]. It has unique and incomparable chemical-physical properties, and it is characterized by numerous biological functions [127]. Hyaluronic acid is an extremely versatile, natural organic compound, non-toxic, biocompatible, biodegradable, and mucoadhesive. Also, hyaluronic acid has excellent antioxidant, good viscoelastic property, excellent gelling property, anti-inflammatory property, wound-healing activity, excellent cosmetic property, and drug carrier ability, therefore, widely be used in the pharmaceutical industry, cosmetic production industry, and biomaterials production industry (Table 1). Also, hyaluronic acid has recently been explored as a drug-delivery agent via different ways such as nasal, oral, pulmonary, ophthalmic, topical, and parenteral [128].

The medical, biomedical, and other applications of hyaluronic acid have been well described and presented; however, after a literature survey, it can be concluded that the recently made progress of grafted, chemically modified, physically blended, and chemical cross-linked hyaluronic acid-based biomaterials need to be the review. Therefore, in this review, we examine the modified forms of hyaluronic acid cross-linked polymers. From analyzing recent reports, it can be concluded that the selection of bio-based monomers, structures, concentrations, and functional groups applied via chemical and/or physical modification methods has a significant effect on natural polymers.

### Biomedical applications of hyaluronic acid

Recently, Hamlet et al. [129] developed novel tissue-regenerative 3D hydrogels by hyaluronic acid cross-linking of medical polycaprolactone (mPCL) containing osteoblasts (OB) and bone morphogenetic protein-7 (BMP-7) for bone tissue engineering. The mPCL-hyaluronic acid hydrogels possess tremendous promise as a supportive network for bone tissue that is bone-like, and thus creates a favorable environment for bone tissue engineering. Hyaluronic acid is a water-soluble bio-based monomer and forms a highly viscous solution. Synovial fluid and vitreous humor have a large quantity of hyaluronic acid that contributes to these tissues viscoelastic properties [130]. It also plays an important structural role in articular cartilage and skin. Moreover, this nonsulfated glycosaminoglycan, which is present in all connective tissue, is a major constituent of the extracellular matrix (ECM) and is particularly prevalent during wound healing. Thus, hyaluronic acid has been proposed for the preparation of biodegradable ECM-like constructs for tissue engineering applications [131]. It has been reported that cross-linking of hyaluronic acid forms experimentally controllable hydrogels that provide a microstructure similar to the native ECM [132]. Indeed, it possesses several properties that make it unique, and thus hyaluronic acid-based biomaterials can be used for biomedical applications.

Kutlusoy et al. [133] recently synthesized natural cryogel scaffolds of chitosan-co-hyaluronic acid with glutaraldehyde as a cross-linker via cryogelation. The prepared hyaluronic acid-based cryogel scaffolds were suitable for the development of 3 T3 and SaOS-2 cells. Their MTT assay results show that the hyaluronic acid-based cryogel scaffolds increased cell proliferation according to an increase in the hyaluronic acid content. Importantly, the hyaluronic acid-based chitosan-co-hyaluronic acid cryogels were not significantly cytotoxic toward 3 T3 fibroblast and SaOS-2 cells. In particular, the mechanical and biomaterial properties of pure chitosan were improved in the copolymers with hyaluronic acid at different concentrations.

Cryogels can be developed for biomedical applications by using natural or synthetic polymers that are excellent biomaterials because they are non-toxic, biocompatible, and biodegradable and allow cell adhesion, growth, and transport [134]. Hyaluronic acid plays an important role in the maintenance of joint lubrication in the hydration and moisturization of tissues, the transition of matter from tissues, as well as the movement, differentiation, and division of cells [135, 136]. It is also the most preferred natural filling material since there is no deformation in structure or shape in the injected place over a long period [137]. Therefore, hyaluronic acid monomer can be used to prepare interconnected macroporous chitosan cryogels that are capable of cell penetration.

Cerqueira et al. [138] successfully developed poly (lactic-co-glycolic acid) (PLGA) nanoparticles embedded with paclitaxel and coated with hyaluronic acid (HA-PTX-PLGA). The HA-PTX-PLGA nanoparticles showed excellent sustained drug release and less PTX cytotoxicity in MDA-MB-231 cells compared to cells incubated with non-hyaluronic acid-coated nanoparticles. Moreover, HA-PLGA nanoparticles exhibited improved cellular uptake possibly based on receptor-mediated endocytosis due to the interaction between hyaluronic acid and CD44 receptors when compared to non-coated PLGA nanoparticles. Hyaluronic acid is desirable as a targeting moiety for PLGA nanoparticles because it is a naturally occurring polyanionic polysaccharide composed of N-acetyl-D-glucosamine and D-glucuronic acid, a substrate that binds specifically to CD44 receptors that are overexpressed in some tumor types (including triple-negative breast cancers) [138]. The most advantageous properties of hyaluronic acid are its non-toxicity, biocompatibility, biodegradability, and non-immunogenic nature (Table 1). More importantly, hyaluronic acid binds specifically to CD44 receptors resulting in the receptor-mediated endocytosis of PLGA nanoparticles [139]. Another important advantage of using it as a targeting moiety for PLGA nanoparticles is its ability to increase their circulation time by providing them with the necessary characteristics to prevent their uptake by the reticuloendothelial system [140]. Paclitaxel is a poorly soluble antimitotic chemotherapeutic agent that causes tumor cell death by disrupting mitosis, and its conjugation with hyaluronic acid monomer greatly increases its solubility. Furthermore, it has been suggested that by using this method, paclitaxel can be released inside tumor cells by intracellular enzymatic hydrolysis of the ester bond to carry out its activity [141]. Taken together, it appears that the coating of hyaluronic acid on paclitaxel-embedded PLGA nanoparticles is a promising avenue for the targeted delivery of paclitaxel to triple-negative breast cancer cells.

Most recently, Wang and co-workers [142] first synthesized novel hyaluronic acid-doped PEDOT nanoparticles by the method of oxidative chemical polymerization, after which they prepared conductive poly 3,4-ethylene dioxythiophene PEDOT-HA/poly(L-lactic acid) (PLLA) composite films. These films did not show significant cytotoxicity and could better support cell adhesion, survival, and spread than PLLA films, thereby proving their suitability as a biocompatible conductive polymer for biomedical applications. In the past few years, polyheterocyclic families mainly including polyaniline, polypyrrole, and polythiophene have been extensively used in biomedical engineering [143]. Specifically, PEDOT, a polythiophene derivative, is one of the most promising conductive polymers due to its tunable electro-optical properties and a high degree of processability [144–148]. PEDOT possesses higher electrical conductivity and stability than other conducting polymers, and it has been successfully used in biosensors, neural electrodes, nerve grafts, and heart muscle patches [149–152].

Specifically, researchers have noted that negatively charged glycosaminoglycans, an important component of the ECM, can be incorporated into conducting polymers by doping [153]. Hyaluronic acid monomer is one of the most important glycosaminoglycans in the vertebrae body. It has been used to improve the biological functioning of conducting polymers used for wound healing and tissue regeneration, as well as performing a vital role in angiogenesis for the manufacture of synthetic matrices [154, 155]. Another point of interest is that it has been established that pyrrole-conjugated hyaluronic acid coating has the potential to mask conducting electrodes from adverse glial responses that occur upon implantation [156]. Similar to other conducting polymers, PEDOT also needs a balancing counterion as a dopant for its polymerization, although there is a lack of information about the mechanism of chemical doping with hyaluronic acid. Nevertheless, the doping of PEDOT-PLLA films with hyaluronic acid is an attractive candidate for the electrical stimulation required to enhance nerve regeneration in nerve tissue engineering.

Recently, Palmieri and co-workers [157] reported a simple approach for developing non-toxic, biodegradable, and biocompatible nanoporous hyaluronic acid microparticles (NPHAs) with characteristic sponge-like morphology and uniform size. The NPHAs were synthesized using the concomitant cross-linking of hyaluronic acid and cross-linking agent precipitation. The developed NPHAs can remain for a long time in vivo without any side-effects and exhibited the sustained release of hyaluronic acid. Hyaluronic acid offers many advantages when used in biomaterials for biomedical applications, including its non-toxicity, biodegradability, biocompatibility, and bioresorbability (Table 1). It is a major intracellular component of connective tissue, where it plays an important role in lubrication, cell differentiation, and cell development, making it especially attractive for bioscaffolds. Hyaluronic acid contains multifunctional groups (carboxylic and alcohol) along its backbone that can be used to introduce functional domains or to form hydrogels via cross-linking [137]. Therefore, the chemical modification of hyaluronic acid monomer has been used to make many versatile polymers for new drug-delivery systems. Because of its favorable physical-chemical and biological features, it has various biomedical uses, such as osteoarthritis treatment, ocular and plastic surgery, and tissue engineering [158]. In vivo studies have recently shown that the half-life of high molecular weight or cross-linked hyaluronic acid preparations can be extended by up to 48 h [159].

The rapid exhaustion of hyaluronic acid formulation from the joint cavity causes pain in osteoarthritis patients. As a result, it is necessary to provide long and stable pain relief with many injections of drugs per year. However, the injected patient often has an increased risk of inconvenience and infection. Thus, hyaluronic acid-based nanoporous microparticles may provide innovative and safe biomaterials to advance osteoarthritis treatment by prolonging the favorable effects of hyaluronic acid for an unprecedented duration after injection into a joint [157].

## Glutamic acid

Glutamic acid is a biodegradable natural bio-based amino acid monomer that is produced by microbial fermentation. It was first discovered and identified in the year 1866 by the German chemist Karl Heinrich Ritthausen who treated wheat gluten (after which it was named) with sulfuric acid [160]. In 1908, mass-production of the crystalline salt of glutamic acid, monosodium glutamate, was reported and patented by Japanese researcher Kikunae Ikeda of the Tokyo Imperial University [161, 162]. Glutamic acid plays an important role in the body’s disposal of excess or waste nitrogen. It undergoes deamination, an oxidative reaction catalyzed by glutamate dehydrogenase [163]. Due to its non-toxicity, biodegradability, biocompatibility, and excellent cation chelating ability, it is widely used in the pharmaceutical, cosmetics, food, water-treatment, agriculture, and other industries (Table 1). Poly (glutamic acid) (PGA) is a natural linear polymer formed by the peptide bonds between the α-amino group and the γ-carboxyl group at the end of the glutamic acid side chain. It can be synthesized by bacilli such as *Bacillus subtilis*, and thus can be obtained from fermentation by using bacteria [164]. Due to its excellent bioactive properties, glutamic acid has been widely applied in biomaterials development using chemical and/or physical modification or cross-linking with natural and synthetic polymers [42–52]. Here, we report the most recent progress made in glutamic acid-based modified forms and the opportunities for applying them in biomedical applications.

### Biomedical applications of glutamic acid

Wang [165] developed ε-polylysine-γ-PGA novel composite hydrogels by using EDC (1-ethyl-3-(3-dimethylaminopropyl)carbodiimide) and NHS (N-hydroxysuccinimide) cross-linking. In this system, gelation is attributed to the cross-linking reaction between the amino groups of ε-polylysine and carboxyl groups of γ-PGA. ε-Polylysine is a biomaterial with underlying antibacterial properties, and the novel composite hydrogels showed excellent antimicrobial activity against *Escherichia coli* and *Staphylococcus aureus*. Furthermore, an indirect cytotoxicity assessment indicates that the novel composite hydrogels are non-toxic to the mouse fibroblasts (L929 cell) [166]. In comparison, the antibacterial activity of some antibiotics and antimicrobial drugs become exhausted more quickly after release.

PGA is a naturally occurring homo-polyamide made of D- and L-glutamic acid that is biodegradable, biocompatible, edible, and non-toxic to humans and the environment (Table 1). Its high surface hydrophilicity improves the overall absorption rate, swelling ratio, and mechanical strength of the resulting biomaterials for biological adhesiveness and tissue engineering [167–169]. In the meantime, it can maintain a moist wound bed for effective wound healing. EDC and NHS have been reported to be non-toxic and biocompatible in vitro [170–172], and the EDC-NHS reaction is conducted in aqueous solution. Moreover, the reaction system can be purified via dialysis, thus producing markedly cross-linked and non-toxic composite hydrogel materials. Therefore, the cross-linking of PGA with ε-polylysine can provide potential wound-dressing biomaterials with excellent healing efficacy for biomedical applications.

Hellmers et al. [173] produced γ-PGA-chitosan composite nanoparticles for their application as a carrier for the anti-cancer drug doxorubicin; the encapsulated or surface-bound drug did not lose its bioactivity and the prepared drug-loaded nanoparticles exhibited considerable antiproliferative activity against the human cancer cell line. However, the nanoparticles drug-delivery system suffers from important problems such as low drug loading capacity, low loading efficiency, and poor ability to control the size distribution [174]. Moreover, due to their ability to reduce the toxic side-effects of drugs, little attention has been given to the possible risks of the nanoparticles [175]. Biodegradable polymers are in great demand for the development of novel drug-delivery systems and as promising biomaterials. Therefore, the potential utility of polyelectrolyte-complex vehicles formed using PGA and chitosan as a novel drug-delivery carrier is unprecedented. PGA is an antimicrobial, nonimmunogenic, biodegradable, biocompatible, hydrophilic, and non-toxic biopolymer (Table 1). Apart from this, since PGA plays a role in the regulation of microtubule dynamics in rats, it has been found that it can be biochemically degraded into glutamic acid residues in vivo [176]. Thus, PGA is a promising environmentally friendly biomaterial for biomedical applications [52, 177].

Most recently, Ma and co-workers [178] developed a triple-responsive zwitterionic hydrogel composed of equal portions of L-glutamic acid and L-lysine polypeptide for site-specific drug delivery. The prepared hydrogels showed high resistance to non-specific protein adsorption and attachment of HUVEC cells, along with good biocompatibility and response to pH and ionic-strength changes. In addition, when doxorubicin and diclofenac were loaded into the hydrogel as model drugs, responsive drug release to both pH and enzyme was demonstrated. In some previous studies, hydrogels prepared with zwitterionic materials, such as poly (sulfobetaine methacrylate) (PSBMA) and poly (carboxy betaine methacrylate) (PCBMA), have attracted great attention due to their ultra-low fouling and extremely biocompatible properties [179–181]. For example, Jiang’s group [182] prepared zwitterionic material-based hydrogels that can resist the formation of capsules for at least three months after subcutaneous implantation in rats. Nevertheless, these materials are problematic due to their non-biodegradability, which can cause side-effects after drug release. Hence, synthetic biodegradable hydrogels are desired for local drug delivery. Hydrogels formed from L-glutamic acid and L-lysine polypeptides can be digested and/or degraded by proteinases. In fact, matrix metalloproteinases, cathepsins, and serine proteases are usually overexpressed which mediates cancer progression and tumor metastasis [183–186]. Therefore, it is possible to use L-glutamic acid and L-lysine polypeptide hydrogels for responding to proteases in local drug-delivery systems. Therefore, L-glutamic acid and L-lysine polypeptide hydrogels are promising materials for drug-delivery systems.

Hu et al. [187] fabricated a series of copolymer hydrogels with controllable mechanical properties through copolymerization of PEG and γ-PGA via photopolymerization. The developed PEG-PGA copolymer hydrogels showed good ionic and pH sensitivity and did not exhibit acute cytotoxicity. Moreover, these hydrogels inhibit drug release at low pH while promoting it at high pH. The abundant α-carboxyl groups of PGA not only offer reactive sites for further functionalization but also facilitate intermolecular and/or intramolecular association through hydrogen bonding or static charge repulsion depending on the environmental pH; such characteristics point toward its potential application in drug-delivery systems through the gastrointestinal tract in that drug release would be inhibited in the stomach but enhanced in the intestine [188–190]. As a polyelectrolyte, the hydration of PGA may be affected by the environmental ionic strength, which in turn results in ionic strength- and pH-responsive behavior. So far, PGA has been utilized in biomedical applications such as drug/gene delivery vehicles/matrices and tissue engineering biomaterials [191–195]. It has also been reported that glutamic acid can act as a signaling biomolecule involved in the mechanical stimuli-induced process of maintaining chondrocyte functionality [196–198]. Hence, PEG-PGA hydrogels are potentially applicable to the design of pH-dependent drug-delivery systems as well as for use as cartilage tissue engineering biomaterials.

Recently, Tong et al. [199] prepared novel alginate-PGA composite microparticles with a controllable structure via an emulsification/internal gelation method. The prepared alginate-PGA novel composite microparticles showed excellent swelling behavior due to the high hydrophilicity of bio-based PGA material and better thermal stability than alginate. Moreover, cytocompatibility testing of the microparticles in vitro exhibited good biocompatibility with L929 cells. Alginate is a naturally occurring bio-based polymer that due to its polyanionic nature, pH sensitivity, non-toxicity, biocompatibility, and biodegradability is widely used for biomedical and other applications [200]. Due to the unusual polyanionic nature of alginate and PGA, a double-network structure is formed in the composite microparticles due to the ion-chelation interaction between Ca^2+^ and the carboxylate groups of alginate and PGA and the electrostatic interaction between the secondary amine group of PGA and the carboxylate groups of alginate and PGA. In a previous study [201], a natural hydrophilic polymer like PGA has been combined with alginate instead of synthetic superabsorbent polymers to improve the swelling capacity of a combined alginate-PGA hydrogel. In another report [202], a layered hydrogel comprising PGA, sodium alginate, and chitosan has prepared to mimic the stability of natural hydrogels in physiological fluids. Recently, the preparation of highly hydrophilic alginate-PGA microparticles has attracted considerable attention. Core-shell alginate-PGA microparticles have been prepared by dropping an alginate solution directly into a calcium chloride (CaCl_2_) solution and coating the alginate microparticles with PGA [52]. Similarly, to prepare alginate-PGA microparticles, an alginate-PGA mixture solution was directly dropped into a CaCl_2_ solution [203]. Therefore, PGA-based composite microparticles, hydrogels, and scaffolds are useful biomaterials for biomedical and other applications.

## Conclusions

In this review article, we covered how natural bio-based monomer materials are applied in various aspects of biomedical applications. Examples of natural bio-based monomers, such as itaconic acid, succinic acid, citric acid, hyaluronic acid, and glutamic acid that play major roles in the design and development of biomaterials were discussed, as were the innovative approaches to developing natural biopolymer-based biomedical moieties. Moreover, the emergence of combination polymers holds promise for the production of novel biomaterials that possess desired properties for specific biomedical and other applications. These include novel copolymers, hydrogels, scaffolds, blending, composite microparticles, nanoparticles, and nanofibers for use in dental, ophthalmic, wound-healing, cosmetic, pharmaceuticals, drug delivery, food flavoring/preservative, and waste-water treatment. We also looked into the role of natural biopolymers concerning their use in specialty biomedical applications, especially to improve their biocompatibility, biodegradability, and bioactivity, and to reduce their toxicity. Natural bio-based monomers show good applicability for the biomedical industry both in the present and future.

## Data Availability

Not applicable.
